# Microwave-Assisted Hydrogenation of Codeine in Aqueous Media

**DOI:** 10.5402/2012/104975

**Published:** 2012-09-20

**Authors:** F. Taktak, I. Bulduk

**Affiliations:** ^1^Department of Chemical Engineering, Faculty of Engineering, Uşak University, 64200 Uşak, Turkey; ^2^Department of Chemistry, Faculty of Arts and Sciences, Uşak University, 64200 Uşak, Turkey

## Abstract

An efficient one-pot microwave-assisted hydrogenation of codeine was achieved in aqueous solution. This technique is simple, fast, environmentally friendly, and highly efficient. Structure of produced dihydrocodeine was approved by using FT-IR, ^1^H NMR, ^13^C NMR, EIMS, and elemental analysis technique. Its purity analysis was performed by using HPLC and assay analysis was performed by using potentiometric titration methods.

## 1. Introduction 

Dihydrocodeine is a semisynthetic alkaloid that has preventive effects of shortness of breath and cough, as well as used extensively in killing postoperative pain [1]. Importance of dihydrocodeine is increasing day by day as second step of the “analgesic ladder” proposed for the treatment of cancer pain [2–4]. By extending the duration of action of the drug, since dihydrocodeine became available as a slow-release preparation, in this regard, it has begun to replace codeine [5]. Dihydrocodeine is also an important intermediate used in synthesis of other alkaloids such as hydrocodone [6].

A number of methods have been reported to prepare dihydrocodeine. Grew and coworkers have prepared dihydrocodeine by hydrogenation of hydrocodone [7, 8]. However, when compared with the hydrogenation process from codeine, additional steps are incorporated into the process and this ultimately increases the cost for obtaining dihydrocodeine. For hydrogenation of codeine palladium supported on carbon, catalysts are generally studied [6, 9]. In these methods, generally there is a need for excessive amounts of organic solvent for hydrogenation these processes bear risks in terms of health as well as causing environmental pollution. Therefore, Rapoport and Lithotworik used water as a solvent in the hydrogenation process of codeine [10, 11]. Disadvantages of some of these methods are a number of side reactions difficult to control, undesirable side products, long reaction times, and necessity of using a deactivating agent which is connected to the surface of catalytic metal that can minimize isomerisation side reactions of codeine. Microwave-assisted organic synthesis is an invaluable technology for drug synthesis because it often reduces reaction times, typically from days or hours to minutes or even seconds [12–14]. On the other hand, the use of microwave irradiation to accelerate catalytic reactions can be used to obtain good results from low-yielding reactions [15, 16]. With the employment of microwaves in metal catalysed reaction are reduced other unwanted side reactions [17–19]. 

Here, we describe the microwave-assisted synthesis of dihydrocodeine, which achieved reductions in reaction times, higher yields, and cleaner reactions than for the previously reported synthetic processes. Dihydrocodeine was obtained in purity suitable for use in pharmacopy (99.80%) in high efficiency (98%) without using any deactivating agent and using metal catalyst in lower rate.

## 2. Results and Discussion 

### 2.1. Synthesis of Dihydrocodeine

**Scheme 1:**
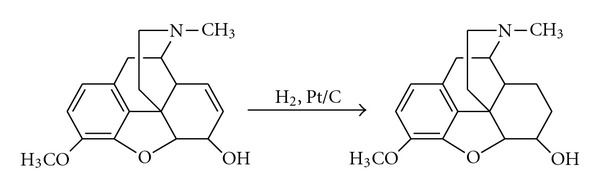
Hydrogenation of codeine to dihydrocodeine.

Synthesis of the dihydrocodeine which is a semi-synthetic opioid was achieved under microwave irradiation out of codeine as outlined in Scheme 1. In this study, an extremely simple, inexpensive, and highly efficient and noncomplex method has been reported. In addition, white crystalline dihydrocodeine in pharmacopical purity is easily obtained alkalinizing the solution environment. Catalyst ratio used is very low when compared with values reported so far; any deactivating agent required to minimize side reactions in reaction environment was not used in these study. Catalyst amount used in selected reaction conditions was determined optimally, the highest efficiency (98%) reported so far in this reaction conditions was obtained, as well as formation of side reactions was significantly eliminated and product in pharmacopy standards was obtained. 

### 2.2. Optimization of Reaction Conditions

In order to determine optimized conditions for the hydrogenation under microwave irradiation, codeine was exposed to different reaction variables. The effect of temperature, reaction time, and microwave power on hydrogenation yield was studied and the initial hydrogen pressure and catalyst ratio fixed as 5 psi and 1% (Pt/C), respectively, in the following experiments. Fixing reaction temperature and time to 20°C and 5 min, respectively, the effect of microwave power on yield of dihydrocodeine was studied. Dihydrocodeine conversion increased from 60 to 98% as microwave power increased from 250 to 450 W, as exhibited in Table 1. Keeping reaction temperature and microwave power to 20°C and 450 W, respectively, the effect of reaction time on the yield of dihydrocodeine was determined. Table 1 indicated that the yield of dihydrocodeine slightly decreased as the irridation time was increased from 5 to 10 min. On the other hand, when the microwave power was set as 350 W, the yield of dihydrocodeine increased as the reaction time was increased from 5 to 10 min. The effect of reaction temperature on dihydrocodeine conversion was investigated. The microwave power and reaction time were set as 450 W and 5 min, respectively. When the temperature was increased from 20 to 40°C, hydrogenation yield decreased significantly. This result was attributed to the fact that side reactions were such as isomerisation of codeine to hydrocodone and cleavage of 4, 5-epoxymorphinane ring of codeine to form dihydrothebainone overly induced at high temperature [11].

Consequently, 20°C, 450 W, and 5 min reaction time are determined as the best conditions in this process. When the temperature and reaction time increased, product yield decreased due to more induced side-reactions. Nevertheless, the reaction efficiency was highly microwave oven power dependent.

### 2.3. Purity Analysis of Dihydrocodeine

**Figure 1:**
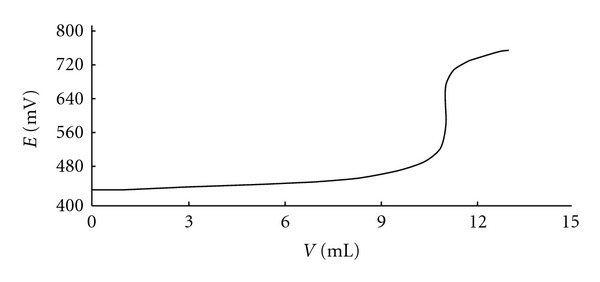
Potentiometric titration curve of dihydrocodeine.

The mV titration curve of this agent is shown in Figure 1. Potentiometric assay showed that dihydrocodeine product conforms to the stated limit as required by the BP [20]. Measurements were repeated five times and the percent purity of dihydrocodeine was calculated as 100.3 on average. 

**Figure 2:**
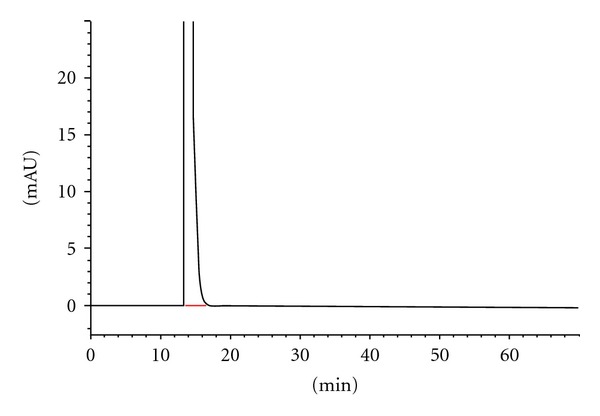
HPLC trace of dihydrocodeine.

The well-exhibited HPLC chromatogram of dihydrocodeine was shown in Figure 2. HPLC of the product revealed the presence of a single peak with a R_t_ of 13.666 min. The chromatographic purity of purified dihydrocodeine was 99.80%.

Finally, it was potentiometrically and chromatographically determined that dihydrocodeine product obtained in the study is in pharmacopy standards. This value complies with the values given in official documents [20].

## 3. Conclusions 

Microwave-assisted preparation procedure stated in this study for dihydrocodeine synthesis offers reduction in the reaction time, operation simplicity, cleaner reaction, easy work-up, and improved yields. All spectroscopic analysis confirmed the proposed structure of this compound.

## 4. Experimental Section 

### 4.1. Materials

All chemicals and platin catalyst (on activated charcoal 1%) used in the present study are of analytical grade purchased from Merck. Codeine in pharmacopy standards was obtained from Turkish Grain Board. 

### 4.2. Characterization Methods

The infrared spectra were recorded as potassium bromide disks using a PerkinElmer Spectrum One FT-IR spectrometer. The ^1^H and ^13^C-NMR spectra of the dihydrocodeine were recorded using the Bruker 500 NMR spectrometer. Mass spectra by EI (electron impact) techniques of dihydrocodeine were determined by an Agilent 6890 model GC-MS. The percentage compositions of the elements (CHNO) for the compound were determined using a ThermoFinnigan FLASH 1112 SERIES EA instrument. 

HPLC analysis. HPLC analysis is conducted using Agilent 1200 which is equipped with UV/VIS Detector. Separation was accomplished on a 25 cm Eurospher-100 (WATERS) C18 column (4.6 mm i.d., 5 *μ*m particle size). The UV detector was set at 284 nm and the flow rate was 1.0 mL/min. The mobile phase was prepared in accordance with the BP method for dihydrocodeine [20].

### 4.3. Potantiometric Measurements

The assay for dihydrocodeine product was determined by potentiometric titration. The potentiometric measurements were made at 25 ± 1°C, using a Mettler Toledo DL53 titrator. 0.35 g dihydrocodeine product was dissolved in 60 mL of glacial acetic acid. This solution was titrated against 0.1 M perchloric acid. The end-point was determined potentiometrically. These measurements were repeated five times. 

### 4.4. Hydrogenation of Codeine

2.5 g codeine was suspended in water (30 mL) and acetic acid was added until codeine dissolved (1 mL). pH of codeine solution was recorded at 4.75. 0.20 g Pt on activated charcoal catalyst (1% Pt/C) was added to solution. This solution was put into a CEM discover microwave reactor (CEM Corporation, Matthews, NC). The upper part of the reactor was filled with inert nitrogen gas by passing nitrogen gas and the addition of H_2_ to the system is started. The initial hydrogen pressure was set as 5 psi in all experiments. H_2_gas was deflated giving the reactor nitrogen gas. The reaction mixture was filtered (0.45 *μ*m of pore size). Active Pt catalyst (on carbon) was removed. Dihydrocodeine was precipitated between 9.80 and 9.90 pH by adding the 30% NaOH solution. Dihydrocodeine was filtered and washed with demineralised water. It was dried in an oven at 105°C. The resulting white solid corresponded to a yield of 98% and purity (by HPLC) of 99.80%. m/z (M+H)+ = 301. ^1^H NMR (500 MHz, d_6_-DMSO): *δ* = 0.90 (q, *J* = 12.2 Hz, 1H), 1.17 (q, *J* = 13.5 Hz, 1H), 1.35 (m, 1H), 1.37 (m, 1H), 1.44 (d, *J* = 11.8 Hz, 1H), 1.82–1.76 (m, 1H), 2.05 (d, *J* = 12.0 Hz, 1H), 2.11 (m, 1H), 2.25 (s, 3H), 2.29 (d, *J* = 5.5 Hz, 1H), 2.34 (d, *J* = 7.6 Hz, 1H), 2.86 (bd, *J* = 18.3 Hz, 1H), 2.93 (bs, 1H), 3.42 (s, 1H), 3.76 (s, 3H), 4.47 (dd, *J* = 18.8–4.4 Hz, 1H) 6.53 (d, *J* = 8.0 Hz, 1H, 6.68 (d, *J* = 8.0 Hz, 1H); ^13^C NMR (500 MHz, d_6_-DMSO): *δ* = 19.32 (CH_2_), 19.58 (CH_2_), 25.75 (CH_2_), 37.12 (CH_2_), 38.29 (CH), 41.80 (q), 42.68 (CH_3_), 45.96 (CH_2_), 56.16 (OCH_3_), 58.81 (CH), 65.89 (CH), 90.31 (CH), 113.87 (CH), 117.95 (CH), 127.11 (q), 130.31 (q), 140.78 (q), 146.96 (q). Anal calcd. for C_18_H_23_NO_3_: C, 71.64; H, 7.62; N, 4.64; found: C, 71.62; H, 7.64; N, 4.40. 

## Figures and Tables

**Table 1:** Optimization of microwave-assisted hydroganation conditions.

Experiment	Temperature	Power (W)	Time (min)	Yield (%)
1	20	250	5	60
2	20	350	5	78
3	20	350	10	84
4	20	450	5	98
5	40	450	5	79
6	20	450	10	93

## References

[1] Edwards JE, McQuay HJ, Moore RA (2000). Single dose dihydrocodeine for acute postoperative pain. *Cochrane Database of Systematic Reviews*.

[2] World Health Organization (1986). *Cancer Pain Relief*.

[3] Leppert W (2010). Dihydrocodeine as an opioid analgesic for the treatment of moderate to severe chronic pain. *Current Drug Metabolism*.

[4] Wang J, Zou J, Gao Y (2005). Double-blinded, controlled, randomized study of dihydrocodeine tartrate vs codeine phosphate in treating cancer pain. *Chinese-German Journal of Clinical Oncology*.

[5] Wotherspoon HA, Kenny GNC, McArdle CS (1991). Analgesic efficacy of controlled-release dihydrocodeine. A comparison of 60, 90 and 120 mg tablets in cold-induced pain. *Anaesthesia*.

[6] Black TH, Forsee JC, Probst DA (2000). A rapid, nearly quantitative conversion of codeine to hydrocodone. *Synthetic Communications*.

[7] Grew EL, Pawles DJ Manufacture of 1-Dihydrocodeine.

[8] Grew EL, Robertson AA Process for the production of dihydrocodeine.

[9] Wieland H, Koralek E (1923). Einige Bemerkungen zur Konstitution des Morphins. *Justus Liebigs Annalen der Chemie*.

[10] Rapoport H, Naumann R, Bissell ER, Bonner RM (1950). The preparation of some dihydro ketones in the morphine series by Oppenauer oxidation. *Journal of Organic Chemistry*.

[11] Likhotvorik I Preparation of dihydrocodeine from codeine.

[12] Ma YM, Zhou X, Wei XY, Zong ZM (2010). The microwave-assisted hydrogenation of 9,10-diphenylanthracene over Pd/C. *Energy Sources, Part A*.

[13] Kappe CO, Dallinger D (2009). Controlled microwave heating in modern organic synthesis: highlights from the 2004–2008 literature. *Molecular Diversity*.

[14] Kappe CO, Dallinger D, Murphree SS (2009). *Practical Microwave Synthesis for Organic Chemists: Strategies, Instruments, and Protocols*.

[15] Vanier GS (2007). Simple and efficient microwave-assisted hydrogenation reactions at moderate temperature and pressure. *Synlett*.

[16] Wang TX, Zong ZM, Zhang JW (2008). Microwave-assisted hydroconversions of demineralized coal liquefaction residues from Shenfu and Shengli coals. *Fuel*.

[17] Banik BK, Barakat KJ, Wagle DR, Manhas MS, Bose AK (1999). Microwave-assisted rapid and simplified hydrogenation. *Journal of Organic Chemistry*.

[18] Barge A, Tagliapietra S, Tei L, Cintas P, Cravotto G (2008). Pd-catalyzed reactions promoted by ultrasound and/or microwave irradiation. *Current Organic Chemistry*.

[19] Desai B, Danks TN (2001). Thermal- and microwave-assisted hydrogenation of electron-deficient alkenes using a polymer-supported hydrogen donor. *Tetrahedron Letters*.

[20] British Pharmacopoeia Commission (2011). *The British Pharmacopoeia 2011*.

